# *In vitro* Dissolution Testing and Pharmacokinetic Studies of Silymarin Solid Dispersion After Oral Administration to Healthy Pigs

**DOI:** 10.3389/fvets.2022.815198

**Published:** 2022-03-01

**Authors:** Ying Xu, Jie Li, Bing He, Tingsong Feng, Lijie Liang, Xianhui Huang

**Affiliations:** College of Veterinary Medicine, South China Agricultural University, Guangzhou, China

**Keywords:** silymarin, solid dispersion, dissolution, pharmacokinetic, pigs

## Abstract

We evaluated the pharmacokinetics of silymarin solid dispersion in pigs to determine whether silybin bioavailability would be increased over that of a silymarin premix. *In vitro* dissolution testing was conducted using dissolution apparatus 1 (baskets) at 100 rpm at 37 ± 0.5°C in pH 1.2 HCl, pH 6.8 phosphate, and pH 4.3 acetate buffers containing 0.5% Tween-80. *In vivo* pharmacokinetics were studied using 16 healthy pigs (Yorkshire × Landrace) that were randomly assigned to two groups. Silymarin as solid dispersion and premix dosage forms were administered directly by stomach tubes at 50 mg kg^−1^ silybin. *In vitro* dissolution of silybin for the premix was 35.02, 35.90, and 38.70% in these buffers, respectively. In contrast, silybin dissolution in solid dispersions was increased to 82.92, 87.48, and 99.70%, respectively. Silymarin solid dispersion administered at a single dose resulted in a peak concentration (C_max_) of 1,190.02 ± 246.97 ng ml^−1^ with the area under the curve (AUC_0−∞_) at 1,299.19 ± 67.61 ng ml^−1^ h. These parameters for the premix groups were 411.35 ± 84.92 ng ml^−1^ and 586.82 ± 180.99 ng ml^−1^ h, respectively. The C_max_ and AUC_0−∞_ values for the solid dispersion were about twice that of the premix and were consistent with the *in vitro* dissolution data.

## Introduction

Silymarin represents a complex polyphenolic mixture extracted from the seeds of the milk thistle *Silybum marianum* L (1, 2) and is composed of 65–80% flavonolignans silybin, isosilybin, silychristin, and silydianin with trace amounts of flavonoids and 20–35% fatty acids and polyphenolics (3, 4). The primary component of silymarin is silybin (CAS No. 22888-70-6), and this has been identified as its bioactive component (4–6). The main component of silymarin is silybin, synonymous with silibinin and is a mixture of two diastereomers A and B in an ~1:1 proportion (6–8).

Silymarin extracts have antioxidant, radical scavenging, and metal chelating activities *in vitro* and *in vivo* that can inhibit neutrophil migration and reduce edema at sites of inflammation (9, 10). Additionally, its flavonolignan and flavonoid compounds have been shown to inhibit JFH-1 virus-induced oxidative stress (11). Interestingly, silymarin has potent effects against hepato-trophic viruses as well as those with other host ranges and is therefore a potential broad-spectrum antiviral (12, 13). Silymarin has an effect that allows its use in all of the most frequent causes of liver damage and possesses three important activities: anti-inflammatory, antioxidant, and pro-apoptotic that can antagonize the onset and the progression of mechanisms of damage that are responsible for the progression of hepatitis to cirrhosis and hepatocellular carcinoma (14).

Silymarin has been examined for veterinary use because animal production processes in modern animal husbandry places a pathological burden on the liver due to disease and side effects of drugs, especially in the piglet stage before fattening (15). Liver damage is a frequent microscopic finding in cases of postweaning multisystemic wasting syndrome (PMWS), and hepatocytes are a target cell for porcine circovirus type 2 (PCV-2) infection and replication (16). Drug-induced bile duct injury in most affects the biliary epithelium of interlobular ducts, and amoxicillin/clavulanic acid was among the most frequent causes of drug-induced liver injury (17). Some drugs can cause chronic liver damage and even lead to tumor growth (18). Silymarin has been classified as a liver therapeutic agent by the WHO (19), and this has attracted the attention of veterinarians. It has been tested in dairy cows suffering from fatty liver during peripartum, and its effects include reduced ketosis and improved lactation performance (20). *S. marianum* incorporated into fodder was a successful substitute for fodder antibiotics in pigs, and weight gains increased and were 6.1% greater compared with the group receiving conventional prophylactic antibiotics (21, 22). Silymarin has also been shown to promote bile secretion and has a choleretic effect, but this was species-specific and also linked to dosage (23).

Silymarin has important biological activities and is currently used in veterinary practices to protect the liver, although its pharmacokinetics in pigs and other production animals has not been fully elucidated. In fact, poor solubility and low bioavailability of silybin limits its therapeutic potential (24). Therefore, further optimization and characterization of *in vitro* dissolution and drug-release profiles of silymarin would be required before it could be used clinically *in vivo*. In the present study, we examined the *in vitro* dissolution profiles of silymarin solid dispersions and premix and assessed their pharmacokinetics and bioavailabilities in pigs.

## Materials and Methods

### Chemicals and Reagents

Silybin reference standard (96.3%) was provided by the China Institute of Veterinary Drugs Control (Beijing, China). Silymarin solid dispersion (see Table 1) was developed by the College of Veterinary Medicine, South China Agricultural University (Guangzhou, China), and produced in a pilot project at Jinhe Bio-Tech (Hohhot, China). Silymarin active pharmaceutical ingredient (API) was calculated as silymarin at 60.25%. Silymarin API, silymarin premix, and pharmaceutical excipients of the premix were all provided by Guangzhou Leader Bio-Technology (Guangzhou, China). Assays of silybin in silymarin solid dispersion and silymarin premix were 3.92 and 3.10%, respectively. Pharmaceutical excipients of silymarin solid dispersion were provided by Jinhe Bio-tech. Acetonitrile and methanol [high-performance liquid chromatography (HPLC) grade] were purchased from Fisher Scientific (Pittsburg, PA, USA). Water was purified using a Milli-Q Water Purification system (Milford, MA, USA). Other chemicals used were of analytical grade and purchased from Damao Chemical Reagent Factory (Tianjin, China).

**Table 1:** Relative composition of the optimal silymarin solid dispersion formulation^a^.

**Ingredient**	**PEG 6000**	**Alcohol**	**Silymarin API^b^**	**Light calcium carbonate**
Composition (%)	58.4	5	16.6	20

*^a^Specification of solid dispersion that was developed was 10% silymarin and 3.92% silybin*.

b *Containing 60.25% silymarin*.

### Animals and Feeding

Ethical approval for *in vivo* experiments in pigs was obtained from the animal ethics committee of South China Agriculture University. Animals used in this study consisted of 16 healthy pigs (Yorkshire × Landrace) weighing 13.68 ± 0.57 kg. The animals were acclimatized in pens for 1 week and given standard commercial feed twice a day with water available *ad libitum*.

### *In vitro* Dissolution

The dissolution study was carried out according to the Chinese Veterinary Pharmacopeia (CVP, 2015) basket method using 900 ml of the following dissolution media: pH 1.2 HCl, pH 4.3 acetate, and pH 6.8 phosphate buffers each containing 0.5% Tween-80. The temperature of the dissolution medium was controlled at 37 ± 0.5°C, and stirring speed was maintained at 100 rpm. Samples (5 ml) were withdrawn at 20, 40 min and 1, 2, 4, and 6 h and replenished immediately with 5 ml dissolution medium. Samples were passed through 0.22 μm filters prior to HPLC quantification. Data at each time point were presented as mean of triplicate samples.

### Pharmacokinetic Experiments

Pigs were randomly divided into two groups, with eight pigs per group, and received solid dispersion and premix by oral gavage at a single dose of silybin 50 mg kg^−1^, respectively. Blood samples (4 ml) were taken from the superior vena cava by syringe and transferred to polyethylene tubes containing heparin at 0, 5, 10, 15, 20, 30, and 45 min and at 1, 1.5, 2, 3, 4, 6, and 8 h post-administration. Plasma samples were obtained by centrifuging the collected blood samples at 4,000 rpm for 10 min and were stored at −20°C.

### HPLC Conditions

Silybin levels in plasma samples were determined using HPLC using an LC-20AT quaternary pump and SPD-20A UV detector (Shimadzu, Kyoto, Japan). Chromatographic separation was achieved on a InertSustain C18 analytical column (250 × 4.6 mm, 5 μm) (Phenomenex, Torrance, CA, USA) using an isocratic mobile phase of 50% methanol in water at 0.9 ml min^−1^ using a detection wavelength of 288 nm.

### Plasma Sample Pretreatment

Plasma samples were processed according to previously published protocols (25). In brief, 1 ml of plasma in 10 ml centrifuge tubes was combined with 1 ml of tert-butyl methyl ether and vortexed for 2 min and then centrifuged at 5,000 rpm for 5 min. A supernatant sample was evaporated under a nitrogen stream in a heating block at 50°C, the residue was reconstituted with 200 μl methanol and passed through a 0.22-μm filter, and 10 μl was used for HPLC injection.

### Pharmacokinetic and Statistical Analysis

The cumulative release (%) of the drug at each time point was calculated according to the following formula:


$$\begin{array}{l} Q\%=\frac{C_{n}\times V+\sum_{i=1}^{n-1}C_{i}\times V_{i}}{W\times DL}\times 100\%,(V_{0}=0,\;C_{0}=\;0) \end{array}$$

where *C*_*n*_ is the concentration of silybin in the sample taken at time *n, V* is the total volume of the release medium, *V*_*i*_ is the sampling volume at time *i, C*_*i*_ is the concentration of the sample taken at time *i, W* is the weight of the solid dispersions, and *DL* is the drug loaded in the solid dispersions.

Silybin concentration–time data were analyzed using WinNonlin 5.2.1 software (Pharsight, Mountain View, CA, USA) using a non-compartment model with best fitting. The pharmacokinetic parameters are presented as mean ± SD and were compared for statistical significance using independent Student *t*-tests. A value of *p* < 0.05 was considered statistically significant. All statistical analyses were performed using SPSS 22.0 statistical software (IBM, Chicago, IL, USA).

## Results

### Method Validation

The method used in this work was validated by adding silymarin to blank excipients from the solid dispersions and premix to 0.5, 1, and 10 μg ml^−1^ silybin. The samples were subjected to the extraction process, and the recoveries for silybin from both blank excipients were 98.67–100.93% and 98.26–101.61%, respectively. Correspondingly, the relative standard deviations ranged from 0.26 to 0.74% and 0.07 to 0.79% respectively. Silybin regression data calculated from spiked samples gave recoveries of 94.16–100.00% in plasma at 30, 100, and 1,000 ng ml^−1^. The intraday and inter-day variations ranged from 2.76 to 9.07% and 6.62 to 8.85%, respectively. The calibration curves were linear in the range of 30–5,000 ng ml^−1^ (*r*^2^ > 0.99). The limit of detection (LOD) was <15 ng ml^−1^, and the limit of quantification (LOQ) was <30 ng ml^−1^.

### *In vitro* Release

**Figure 1:**
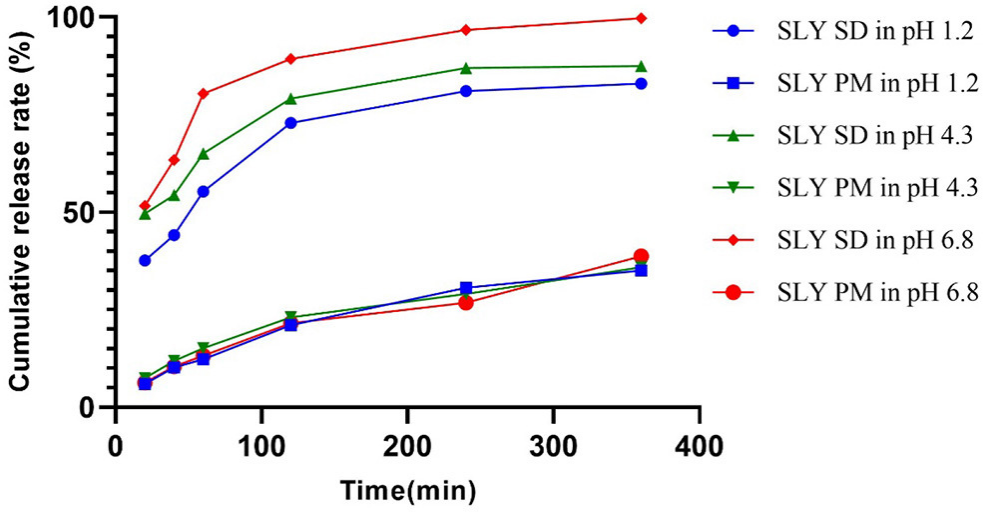
*In vitro* cumulative release curves of silymarin solid dispersion and silymarin premix in different pH buffer solutions. SD, solid dispersion; PM, premix; SLY, silymarin.

The total accumulated amount of the solid dispersion that was released was >80% silybin at pH 1.2, 4.3, and 6.8 in 0.5% Tween 80 solutions, respectively. In contrast, the maximum released by the silymarin premix was only 38.70% in these three buffers, respectively. Overall, the cumulative release rate of silymarin solid dispersion exceeded that of the premix and the total released per unit time was 2.5-fold higher (Table 2, Figure 1).

**Table 2:** *In vitro* silymarin cumulative release for solid dispersion and premix in the indicated buffers.

**Time (min)**	**pH 1.2 HCl**		**pH 4.3 acetic acid**		**pH 6.8 phosphate**	
	**Silymarin solid dispersion**	**Silymarin premix**	**Silymarin solid dispersion**	**Silymarin premix**	**Silymarin solid dispersion**	**Silymarin premix**
20	37.65	5.95	49.63	7.42	51.65	6.30
40	44.10	10.24	54.35	11.86	63.40	10.45
60	55.30	12.32	65.02	15.14	80.40	13.20
120	72.90	21.00	79.16	23.02	89.25	21.55
240	81.00	30.59	86.93	29.05	96.70	26.75
360	82.92	35.02	87.48	35.90	99.70	38.70

### Pharmacokinetics in Pigs

**Figure 2:**
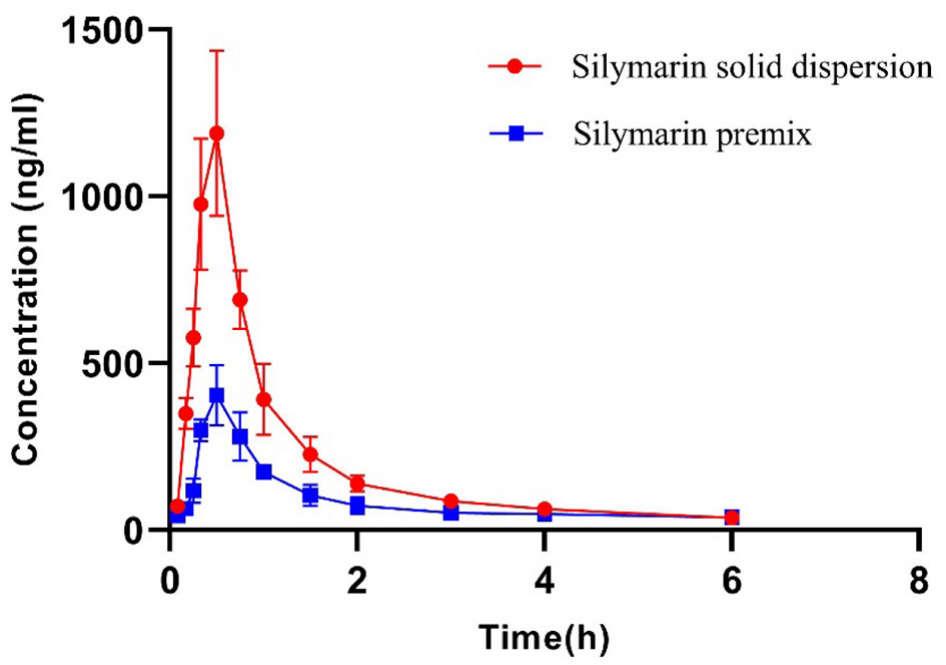
Plasma concentration–time curves of silymarin after single-dose administration at a dose of 50 mg kg^−1^. Data represent mean ± SD values for eight pigs.

Silybin levels in porcine plasma samples indicated that both formulations possessed the same T_max_ while the peak concentrations for the solid dispersion reached 1,189.26 ng ml^−1^ and were ~3-fold greater than for the premix (Figure 2, Table 3). The solid dispersion raised the AUC_0−∞_ to 1,299.19 ng ml^−1^ h, and the relative bioavailability increased more than 2-fold (Table 4).

**Table 3:** Silymarin concentration–time data at different time points following administration of solid dispersion and premix to pigs.

**Time (h)**	**Plasma concentration (ng ml**^**−1**^**) X** **±SD (*****n*** **=** **8)**	
	**Solid dispersion**	**Premix**
0.08	72.31 ± 8.68	42.89 ± 7.94
0.17	349.88 ± 47.28	64.54 ± 8.55
0.25	577.49 ± 87.23	117.51 ± 37.08
0.33	976.46 ± 196.87	299.81 ± 33.27
0.50	1,189.26 ± 247.86	404.71 ± 90.65
0.75	691.18 ± 88.68	280.82 ± 73.27
1	392.26 ± 106.50	174.44 ± 17.19
1.5	226.35 ± 53.64	103.95 ± 31.09
2	138.85 ± 23.81	72.82 ± 25.76
3	86.97 ± 18.86	51.27 ± 22.37
4	63.06 ± 11.55	48.29 ±18.57
6	36.62 ± 2.86	38.20 ± 6.19
8	-	-

**Table 4:** Comparison of pharmacokinetic parameters using a non-compartmental model for solid dispersion and premix administered to pigs.

**Parameter**	**Unit**	**Solid dispersion**	**Premix**	***p-*value**
		**X ±SD (*n* = 8)**	**X ±SD (*n* = 8)**	
Kel	h^−1^	0.36 ± 0.09	0.49 ± 0.30	>0.05
t_1/2_	h	2.02 ± 0.47	2.06 ± 1.46	>0.05
T_max_	h	0.48 ± 0.06	0.48 ± 0.06	>0.05
C_max_	ng ml^−1^	1,190.02 ± 246.97	411.35 ± 84.92	<0.05
V_d_	L kg^−1^	112.10 ± 24.90	239.34 ± 119.53	<0.05
AUC_0−∞_	ng ml^−1^ h	1,299.19 ± 67.61	586.82 ± 180.99	<0.05
MRT	h	1.96 ± 0.35	2.63 ± 1.36	>0.05

*Kel, elimination rate constant; t1/2, elimination half-life; Tmax, time to reach maximum concentration; Cmax, maximal concentration in plasma after oral administration; V_d_, apparent volume of distribution; AUC0-∞, area under the plasma concentration vs. time curve from 0 to ∞; MRT, mean residence time. Values are the mean ± SD, n = 8. p > 0.05 and p < 0.05 were calculated using independent t-tests*.

## Discussion

Silymarin is widely used as a liver protectant in veterinary clinics by virtue of its free radical scavenging, anti-oxidative, and anti-lipid peroxidative activities as well as its antifibrotic and anti-inflammatory activities. However, these positive effects cannot be realized unless the drug is released and generally dissolved in the fluids of the gastrointestinal tract (26). The solubility of silymarin in water is only 0.04 mg ml^−1^, and it is not lipophilic; this results in low permeability across intestinal epithelial cells (27–29). Therefore, it was particularly important to modify the dosage form of silymarin to enhance its bioavailability.

Solid dispersion is a preparation technology used to disperse poorly soluble drugs in solid carrier materials to a form that enhances dissolution rates and solubility. This thereby improves absorption and bioavailability (30). Silymarin solid dispersions have been prepared by fusion and solution-enhanced dispersion using the supercritical fluids (SEDS) and the “dripping pills” methods (31, 32). However, spray granulation has proven to be easier and more efficient and can be used for continuous production in manufacturing (33). We found a cumulative release from silymarin solid dispersion prepared in HCl, phosphate, and acetate buffers containing Tween-80 that was about 2.5-fold greater than for the silymarin premix. These data also indicated that the *in vivo* release would also be enhanced, and we verified this in pharmacokinetic experiments using pigs.

**Figure 3:**
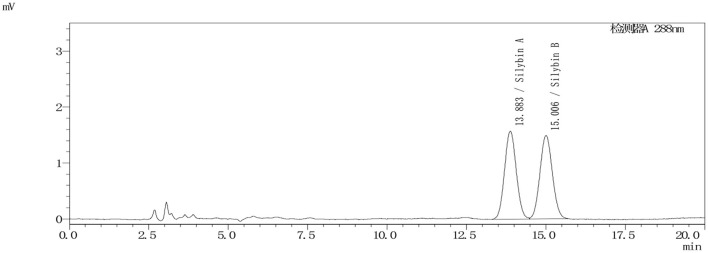
Representative chromatogram of silybin extracted from pig plasma sample at 1,000 ng ml^−1^.

Silymarin has been shown to inhibit acute inflammation in a dose-dependent fashion, and the dose that resulted in 50% inhibition of the *in vivo* inflammatory response (ED_50_) was 62.42 mg kg^−1^, calculated as the amount of silybin present (9). Interestingly, silybin (20–50 mg kg^−1^ day^−1^) has also shown to be effective against amanitin poisoning (34). We therefore administered a comparable ED_50_ of 50 mg kg^−1^ silybin as the single dosage administered orally to pigs. Silybin is isomeric, so we calculated the total amounts using the total areas of peaks that eluted at 14 and 15 min, which was used for pharmacokinetic calculations (see Figure 3) (35).

The rapid and extensive phase II metabolism of silybin has been considered as another reason contributing to its low bioavailability (36, 37). The apparent volumes of distribution we calculated were >1.0 L kg^−1^ and revealed higher tissue levels than plasma levels (38). Peak levels of silybin were observed at 0.5 h in liver, lung, stomach, and pancreas of mice after oral administration at 50 mg kg^−1^ (39). Silybin is primarily excreted in conjugated form through the hepato-biliary tract, and bile levels can exceed plasma levels by 100-fold (24, 40, 41). Silybin can be rapidly and readily transported to tissues after administration, and this would also contribute to its short plasma t_1/2_.

For human volunteers, after oral administration of single doses of 102, 153, 203, and 254 mg, plasma t_1/2_ were <1 h (42). The plasma t_1/2_ of 12.2 min was also obtained using a non-compartmental method after oral administration of 500 mg kg^−1^ to rats (41). A two-compartment model was fitted to silymarin material, and pro-liposome plasma concentrations in beagles generated t_1/2β_ levels of 2.78 and 1.61 h after administration of a dose of 7.7 mg kg^−1^ (35). Short plasma t_1/2_ levels were also independent of animal species. Silymarin is eliminated *via* hepato-enteric circulation, but we did not find double peaks on the drug concentration–time curves (Figure 2).

The silymarin solid dispersion achieved relatively higher peak concentrations and AUC in pigs compared with the premix. Administration of 50 mg kg^−1^ silybin premix generated a C_max_ of 411 ± 85 ng ml^−1^, and the AUC_0−∞_ was 587 ± 181 ng ml^−1^ h in pigs, while the solid dispersion increased these numbers to 1,190 ± 247 ng ml^−1^ and 1,299 ± 68 ng ml^−1^ h. The C_max_ and AUC_0−∞_ for the solid dispersion group were both statistically different (*p* < 0.05) from the premix group. However, no significant statistical difference was observed for pharmacokinetic parameters of solid dispersion such as elimination rate constant (Kel), elimination half-time (t_1/2_), and the peak time (T_max_) when compared to the premix. Meanwhile, the solid dispersion showed higher C_max_ and AUC values than those of premix at the same time point, denoting that the *in vivo* dissolution is the rate-limiting step of *in vivo* absorption and bioavailability due to its high dependency on the drug solubility, namely, the amount of drug in the absorption site (43). The increase in AUC indicated that the developed silymarin solid dispersion improved the absorption of silymarin *in vivo* and was consistent with the *in vitro* dissolution profiles for both these preparations; both increased bioavailability and is a positive factor for reducing costs while increasing efficacy.

New drug development often relies on *in vitro* dissolution experiments because they can predict *in vivo* results based on *in vitro* data. This reduces development time and optimizes the formulation during pharmaceutical development (44). Silymarin is categorized as a class IV compound according to the Biopharmaceutical Classification System (BCS), so the likelihood for *in vivo*–*in vitro* correlations (IVIVC) would be small (45, 46). Nevertheless, we observed that the total amount of silymarin released in dissolution of the solid dispersion in pH 6.8 phosphate buffer solution was × 2.5 that of the premix, and C_max_ and AUC in pharmacokinetics were about × 2.9 and × 2.2 that of the premix group. These proportional changes between pharmacokinetic parameters and amount dissolved *in vitro* indicated that *in vitro* dissolution data may be used to select pilot formulations and quality controls for production.

## Conclusions

This study first showed the pharmacokinetics of silymarin in pigs with rapid absorption and elimination after oral administration. The pharmacokinetic parameters in pigs obtained in the study can be a theoretical foundation for establishing the clinically effective dose regimens of silymarin. The solid dispersion exhibited the higher C_max_ and AUC compared to the premix, and that is consistent with *in vitro* dissolution data. This study provides a basis for further testing of silymarin solid dispersion.

## Data Availability Statement

The original contributions presented in the study are included in the article/supplementary material, further inquiries can be directed to the corresponding author/s.

## Ethics Statement

The animal study was reviewed and approved by the Animal Research Committee of the South China Agriculture University.

## Author Contributions

XH and YX designed and coordinated the project and agreed to be accountable for all aspects of work ensuring integrity and accuracy. JL and TF performed the animal experiment and sample detection by HPLC. BH and LL contributed data processing analysis and chart drawing. The manuscript was drafted and critically revised by YX. All authors read and approved the final manuscript.

## Funding

The study was financially supported by the National Key Research and Development Program of China (Grant No. 2016YFD0501306).

## Conflict of Interest

The authors declare that the research was conducted in the absence of any commercial or financial relationships that could be construed as a potential conflict of interest.

## Publisher's Note

All claims expressed in this article are solely those of the authors and do not necessarily represent those of their affiliated organizations, or those of the publisher, the editors and the reviewers. Any product that may be evaluated in this article, or claim that may be made by its manufacturer, is not guaranteed or endorsed by the publisher.
